# Aetiology and outcomes of sepsis in adults in sub-Saharan Africa: a systematic review and meta-analysis

**DOI:** 10.1186/s13054-019-2501-y

**Published:** 2019-06-11

**Authors:** Joseph M. Lewis, Nicholas A. Feasey, Jamie Rylance

**Affiliations:** 1 Liverpool School of Tropical Medicine, Pembroke Place, Liverpool, L3 5QA UK; 2 Malawi Liverpool Wellcome Clinical Research Programme, Blantyre, Malawi

**Keywords:** Sepsis, Africa south of the Sahara, Bloodstream infection, Tuberculosis, HIV

## Abstract

**Background:**

Aetiology and outcomes of sepsis in sub-Saharan Africa (sSA) are poorly described; we performed a systematic review and meta-analysis to summarise the available data.

**Methods:**

Systematic searches of PubMed and Scopus were undertaken to identify prospective studies recruiting adults (> 13 years) with community-acquired sepsis in sSA post-2000. Random effects meta-analysis of in-hospital and 30-day mortality was undertaken and available aetiology data also summarised by random effects meta-analysis.

**Results:**

Fifteen studies of 2800 participants were identified. Inclusion criteria were heterogeneous. The majority of patients were HIV-infected, and *Mycobacterium tuberculosis* was the most common cause of blood stream infection where sought. Pooled in-hospital mortality for Sepsis-2-defined sepsis and severe sepsis was 19% (95% CI 12–29%) and 39% (95% CI 30–47%) respectively, and sepsis mortality was associated with the proportion of HIV-infected participants. Mortality and morbidity data beyond 30 days were absent.

**Conclusions:**

Sepsis in sSA is dominated by HIV and tuberculosis, with poor outcomes. Optimal antimicrobial strategies, including the role of tuberculosis treatment, are unclear. Long-term outcome data are lacking. Standardised sepsis diagnostic criteria that are easily applied in low-resource settings are needed to establish an evidence base for sepsis management in sSA.

**Electronic supplementary material:**

The online version of this article (10.1186/s13054-019-2501-y) contains supplementary material, which is available to authorized users.

## Introduction

Sepsis, defined most recently as a syndrome of life-threatening organ dysfunction due to a dysregulated host response to infection [1], is common worldwide and carries a high mortality: recent estimates suggest 19.4 million yearly cases and 5.3 million deaths [2]. In high-income settings, outcomes are improving, due in part to a comprehensive application of an expanding evidence base for early recognition, rapid administration of appropriate antimicrobials, and aggressive fluid resuscitation paired with intensive monitoring of physiology and provision for organ support [3, 4]. In low-resource settings including sub-Saharan Africa (sSA), data are limited but some studies have identified high mortality [5]. It is clear that sepsis protocols developed in high-income settings should not simply be exported unchanged to sSA: aggressive fluid resuscitation has been shown to be harmful in one randomised controlled trial (RCT) in adults and one in children [6, 7] and caution is warranted before proposing fluid management guidelines for sSA.

The paucity of data presents challenges in proposing sepsis management specific to sSA. Firstly, defining sepsis for clinical practice or research is problematic. Recent Sepsis-3 guidelines (the third iteration of the international consensus diagnostic definitions of sepsis) suggest operationalising the diagnosis of sepsis using the sequential organ failure assessment (SOFA) score [1]. Applying this score to resource-limited settings is difficult due to patchy availability of variables, particularly laboratory values. The bedside “quick SOFA” (qSOFA) score can identify patients at a higher risk of death, but is a screening rather than a diagnostic tool [8–10]. Previous iterations of the guidelines defined sepsis using the systemic inflammatory response score (SIRS) in the presence of a suspicion of infection, with severe sepsis defined by the addition of organ dysfunction [11], but SIRS, while applicable at the bedside, has been criticised for its lack of discriminatory power [12].

Secondly, the optimal clinical management of sepsis in sSA is unknown. Early, appropriate antimicrobials improve outcomes in high-income settings [13, 14], and it is likely that this is a transferable recommendation to sSA. Certainly, it seems unlikely that rapid administration of antimicrobials will adversely affect outcomes in the way aggressive fluid resuscitation does and may represent an important first step in improving outcomes. However, in the absence of robust sepsis aetiology data, what constitutes “appropriate” empirical antimicrobial chemotherapy remains an open question in sSA. Tuberculosis and malaria certainly play an important role, and arboviral infections, bacterial zoonoses, and HIV opportunistic infections may also be important [15–19].

Finally, sepsis outcomes in sSA are heterogeneous between studies [20, 21] and longer-term follow-up is sparse. Post-discharge sepsis mortality in high-income settings is significant (11–43% at 1 year) as is morbidity [22], but post-discharge outcomes in sSA are unknown.

We therefore performed a systematic review and meta-analysis of clinical studies from sSA with three aims: to describe the sepsis definitions used, the aetiology of infection, and clinical outcomes.

## Methods

The protocol of this systematic review was pre-registered on PROSPERO, number CRD42019123589, and follows the PRISMA guidelines.

### Searches

Searches of PubMed and Scopus were undertaken using the search terms shown in Box 1, in all fields. Identified abstracts were exported into Endnote X7.8 (Thomson Reuters, USA) and screened against inclusion and exclusion criteria.

Inclusion criteria were as follows: prospective cohort studies, non-randomised intervention studies, or randomised controlled trials recruiting adults (> 13 years) with community-acquired sepsis from sSA, for which it was possible to disaggregate a total number of adults with sepsis and was possible to extract aetiology or outcome data. Any study-defined definition of sepsis was accepted. Retrospective studies were excluded due to a high risk of bias: we were concerned that in low-resource settings medical records can often be incomplete and this could introduce significant bias. Exclusion criteria were studies published before 2000 and studies recruiting preselected populations, e.g. puerperal sepsis. There was no language restriction.

Abstracts were screened by two authors (JL and JR) and disagreements resolved by consensus. All included abstracts underwent independent full-text review by the same authors, and the inclusion and exclusion criteria were again applied, and disagreements resolved by consensus. Data were extracted onto an Excel spreadsheet (Microsoft, USA) for further analysis: study first author, years of recruitment, inclusion and exclusion criteria, number of patients recruited and demographics (including age, proportion of HIV-infected participants, and CD4 count values), in-hospital, 28- or 30-day mortality, and details of any aetiologic investigations undertaken.

### Quality assessment

A modified Newcastle-Ottawa scale was used to assess risk of bias in the domains of selection, comparability, and outcome (full scale shown in Additional file 1) This was completed by two authors independently, and disagreements were resolved by consensus to provide a single assessment of each study incorporating all domains.

### Statistical analysis

Because of concerns about meta-analysis of proportions on very heterogeneous populations, we planned meta-analysis of outcome stratified by inclusion criteria where possible: Sepsis-2 sepsis, Sepsis-2 severe sepsis, and Sepsis-3 sepsis, if available. Mortality was presented as a simple proportion with exact binomial confidence intervals, and pooled mortality estimates were calculated using generalised linear mixed models (a normal-binomial model) using the packages *meta* and *lme4* in R. For interventional studies, the outcomes in the usual care arm of the study only were included in these estimates. Heterogeneity was quantified with *τ*^2^, *I*^2^, and Cochran’s *Q* test. Exploratory meta-regression was undertaken to explore heterogeneity by including covariates as fixed effects (year of recruitment, proportion of patients infected with HIV, and median age) and testing for improved model fit by likelihood ratio testing of nested models. A *p* value of < 0.05 was considered a statistically significantly improved fit. Bubble plots of the meta-regressions were produced with 95% confidence intervals obtained from 1000 bootstrap replicates. Summary estimates of 28- and 30-day mortality, where available, were considered together and were presented in the same way. Pooled prevalence estimates of malaria, bloodstream infection (BSI), and *Mycobacterium tuberculosis* bloodstream infection (MTB-BSI) were calculated using random effects meta-analysis as above. For these aetiology analyses, we included all studies, regardless of sepsis definition, and included both usual care and intervention arms of RCTs. All analysis was carried out in R V3.5.1 (R Foundation for Statistical Computing, Vienna, Austria).

## Results

**Fig. 1:**
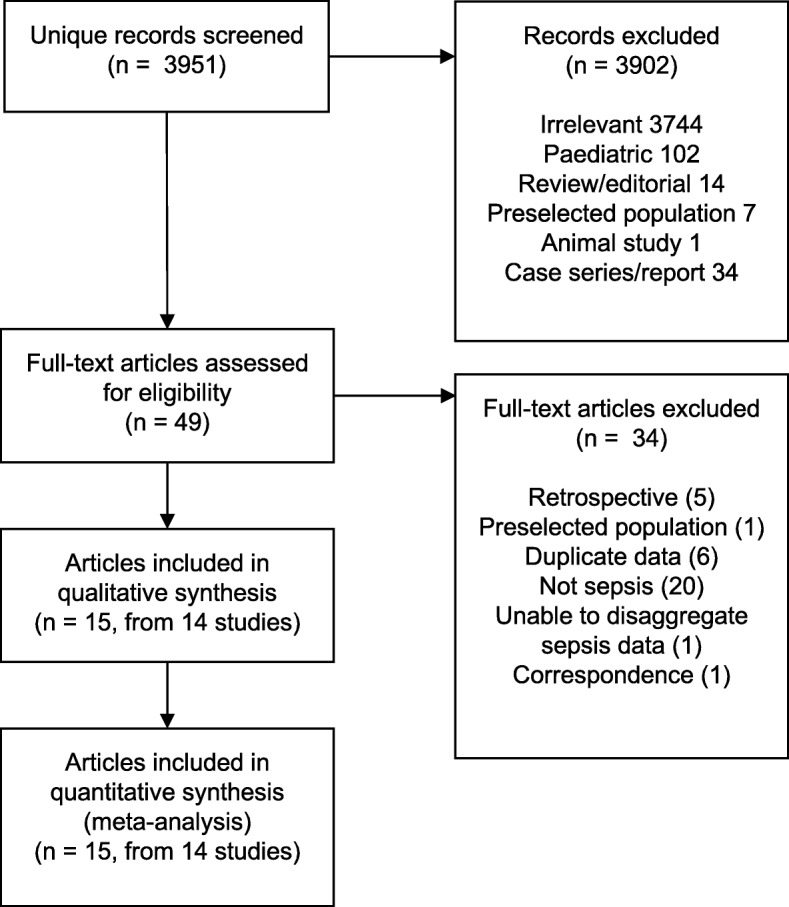
Summary of studies screened and included

The abstract search yielded 3951 unique records on 17 July 2018 (Fig. 1), of which 3902 were excluded. After screening 49 full-text articles, 15 were retained. One article was a secondary analysis of the aetiology of a previously presented cohort [23], meaning 15 articles were included, reporting on 14 prospective clinical studies from nine centres in six countries. These included 11 cohort studies [5, 20, 21, 24–31], two randomised controlled trials [32, 33], and one before-after interventional trial [34] (Table 1).

**Table 1:** Characteristics of included studies

Author and publication year	Years recruiting	Study type	Country	*N* centres	Centre type	Inc. criteria	*n*	Male sex	Age	HIV infected	Median CD4 μL^− 1^
Jacob et al. 2009 [5]	2006	Cohort	Uganda	2	Referral	SII severe sepsis	382	156/382 (41%)	35	320/382 (85%)	52
Nadjm et al. 2012 [25]	2007	Cohort	Tanzania	1	District	Fever and one severity criteria	198	67/198 (34%)	37	69/180 (38%)	NR
Jacob et al. 2012 [34]^a^ and Moore et al. 2018 [23]	2008–2009	Before-after	Uganda	2	Referral	SII severe sepsis	426	207/426 (49%)	34	362/426 (85%)	63
Waitt et al. 2015 [26]	2008–2009	Cohort	Malawi	1	Referral	SII sepsis	213	87/213 (41%)	30	161/213 (76%)	NR
Ssekitoleko et al. 2011 [27]^a^	2009	Cohort	Uganda	1	Referral	SII sepsis	96	193/418^b^ (46%)	35	331/418^b^ (83%)	NR
Ssekitoleko et al. 2011 [28]	2009	Cohort	Uganda	1	Referral	SII sepsis	150	94/150 (63%)	35	96/150 (64%)	NR
Chimese et al. 2012 [21]	2010	Cohort	Zambia	1	Referral	SII sepsis	161	79/161 (49%)	39	110/138 (80%)	NR
Andrews et al. 2014 [35]	2012	RCT	Zambia	1	Referral	SII severe sepsis	112	58/109 (53%)	35	88/109 (81%)	NR
Auma et al. 2013 [29]	2012	Cohort	Uganda	1	Referral	SII sepsis	216	106/216 (49%)	32	122/216 (56%)	NR^e^
Andrews et al. 2017 [33]	2012–2013	RCT	Zambia	1	Referral	SII severe sepsis	209	117/209 (56%)	36	187/209 (90%)	66
Huson et al. 2014 [20]	2012–2013	Cohort	Gabon	1	Referral	SII sepsis	107	NA	34	26/107 (24%)	168
Seboxa et al. 2015 [30]	2012–2013	Cohort	Ethiopia	1	Referral	SII sepsis	292	151/292 (52%)	27	40/209 (19%)	NR
Rudd et al. 2017 [31]	2013	Cohort	Uganda	1	District	SII sepsis	20	11/20 (55%)	32	6/20 (30%)	NR
Amir et al. 2016 [24]	2014–2015	Cohort	Uganda	1	Referral	SII severe sepsis	218	110/218 (50%)	35	125/218 (57%)	78

*RCT* randomised controlled trial, *SII* Sepsis-2 definition (e.g. SII sepsis is consistent with Sepsis-2 definition). ^a^These studies also present data from Jacob et al. [5]—only new data is included in this table row. ^b^Disaggregated data are not given for the included 96 patients; HIV prevalence for the total cohort is shown

Data from 2800 unique participants were eligible (Table 1). Data from some participants were included in two or more separate publications, but we were able to extract aggregate data such that no participant contributed data twice. Generally, the risk of bias as assessed by the modified Newcastle-Ottawa scale was low (Additional file 2). The most commonly identified areas of concern were in ascertaining exposure (all studies ascertained HIV status but 6/14 studies lacked details of HIV testing procedures) or in comparability (5/14 studies did not test lactate and/or provide details of enrollment physiology, both components of the comparability domain of our modified Newcastle-Ottawa score). The patients recruited to the identified studies had median age ranging from 27 to 39 years and were predominantly HIV-infected: 2577/2800 patients had an available HIV status, with 1712/2577 (66%) being HIV-infected, though HIV rates varied between studies (median 70% [IQR 42.5–82.5%], Table 1).

The majority of studies recruited patients using a modified Sepsis-2 definition of sepsis or severe sepsis, though definitions were heterogeneous (primary study inclusion and exclusion criteria are shown in Additional file 3). SIRS was used to define sepsis or severe sepsis in 13/14 cohorts, but the definition of SIRS itself was variable. Six of the 13 studies using SIRS did not use the white cell count criterion because of resource limitations, and four different temperature thresholds were used in the 13 studies to define hypo- or hyperthermia.

Of the 13 studies using SIRS as a component of inclusion criteria, 8/13 recruited patients with a definition compatible with Sepsis-2 sepsis (SIRS plus suspected or confirmed infection, 1255 patients in total) and 5/13 studies recruited a population compatible with Sepsis-2 severe sepsis of SIRS plus organ dysfunction (1347 patients in total). A variety of organ dysfunction criteria were used including hyperlactataemia or poor Karnofsky performance score; the most common organ dysfunction criterion was low systolic blood pressure, applied in all five studies. No study specifically recruited patients with septic shock, and despite the frequent use of low systolic blood pressure as a defining criterion for severe sepsis, only one study [28] explicitly defined septic shock, using a definition of hypotension (SBP < 90 mmHg or MAP < 65 mmHg) refractory to 2 L of intravenous fluid administered over 2 h; 27 of 150 recruited patients fulfilled this definition.

The one study that used a non-Sepsis 2-based definition defined sepsis as fever plus one severity criterion [25], adapted from the WHO severe malaria definitions.

### Outcomes

**Fig. 2:**
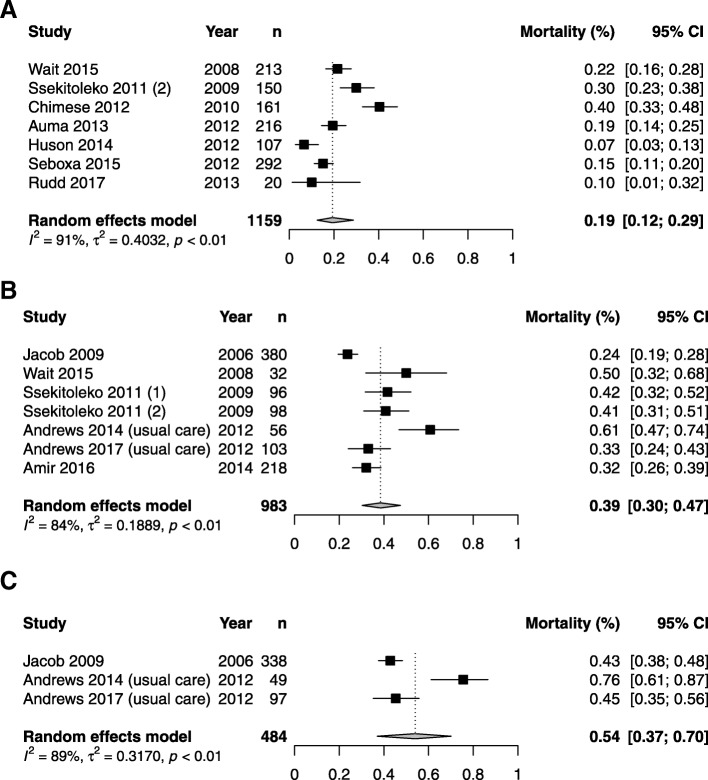
**a** In-hospital sepsis mortality. **b** In-hospital severe sepsis mortality. **c** 28- or 30-day severe sepsis mortality. In all cases, only studies using a Sepsis-2 definition of sepsis or suspected sepsis are included. Random effects summary estimate from a generalised linear mixed model is shown as well as measures of heterogeneity: *τ*^2^, *I*^2^, and *p* value from Cochran’s *Q* test

All studies reported either in-hospital or 28-/30-day mortality, apart from one retrospective of stored samples [23], leaving 14 studies with outcome data. Of these, 11 reported only in-hospital mortality, three studies reported both 28-/30-day mortality and in-hospital mortality, and one reported only 28-/30-day mortality. No studies reported longer-term outcomes or estimates of morbidity. We therefore pooled the outcome data from the 13 studies with outcome data that used Sepsis-2 based inclusion criteria: in-hospital mortality data were available for Sepsis-2-compatible sepsis (1159 participants from seven studies) and severe sepsis (983 participants from seven studies). The 28-/30-day mortality data were only available for severe sepsis (484 patients from three studies). Pooled estimates of Sepsis-2-defined sepsis and severe sepsis in-hospital mortality were 19% (95% CI 12–29%) and 39% (95% 30–47%) respectively, and pooled 28- and 30-day Sepsis-2-defined severe sepsis mortality was 54% (95% 37–70%), though significant between-study heterogeneity means these summary estimates should be treated with caution (Fig. 2).

**Fig. 3:**
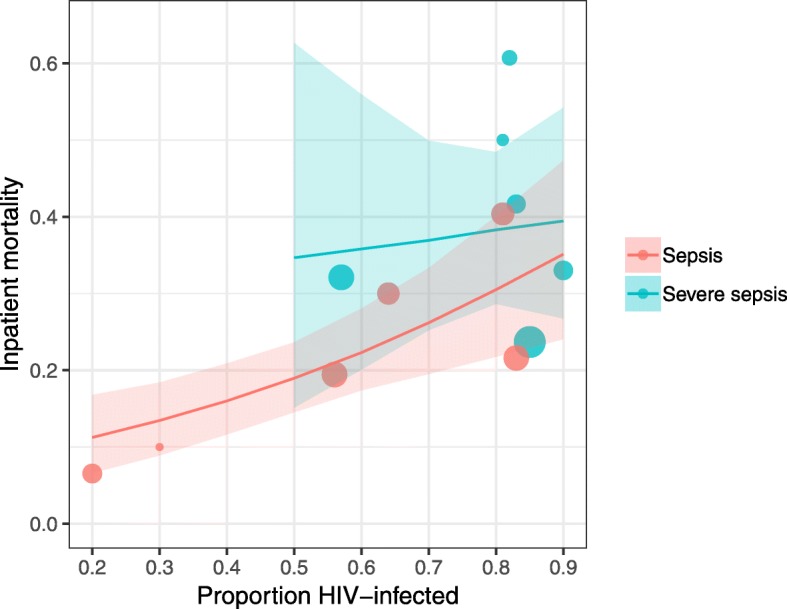
Meta-regression showing the effect of varying proportions of HIV-infected participants on inpatient mortality for sepsis and severe sepsis. 95% confidence intervals generated from 1000 bootstrap replicates. Likelihood ratio testing of nested models shows that including proportion of HIV-infected participants significantly improved model fit for sepsis (*p* = 0.008), but not severe sepsis (*p* = 0.83). Size of circle is proportional to the number of participants with sepsis or severe sepsis

Heterogeneity was explored with meta-regression. Proportion of HIV-infected participants was significantly associated with inpatient mortality in studies recruiting patients with Sepsis-2-defined sepsis when included as a fixed-effect covariate (*p* = 0.006 on likelihood ratio testing of nested models), but year of recruitment was not (*p* = 0.06). Neither year nor proportion of HIV-infected participants (*p* = 0.51 and *p* = 0.83) were associated with inpatient mortality in Sepsis-2 severe sepsis studies. Severe sepsis studies in general appeared to have higher proportion of HIV-infected participants than studies recruiting patients with sepsis (Fig. 3); in only one study [26] was it possible to extract data to compare HIV prevalence in sepsis and severe sepsis in the same study: HIV prevalence was higher in severe sepsis in absolute terms though numbers were small and the difference was not statistically significant (HIV prevalence 26/32 [81%] severe sepsis vs 161/213 [76%] in sepsis, *p* = 0.89). Median age was not associated with in-hospital mortality in sepsis or severe sepsis analyses (*p* = 0.66 and *p* = 0.50 on likelihood ratio testing of nested models). In view of the small number of studies reporting 28- or 30-day mortality, meta-regression was not undertaken.

Outcome data on septic shock were largely absent. For only 27 patients from the only study which defined septic shock was it possible to extract in-hospital mortality: 16/27 (59%, 95% CI 39–77%) patients died.

### Aetiology

**Fig. 4:**
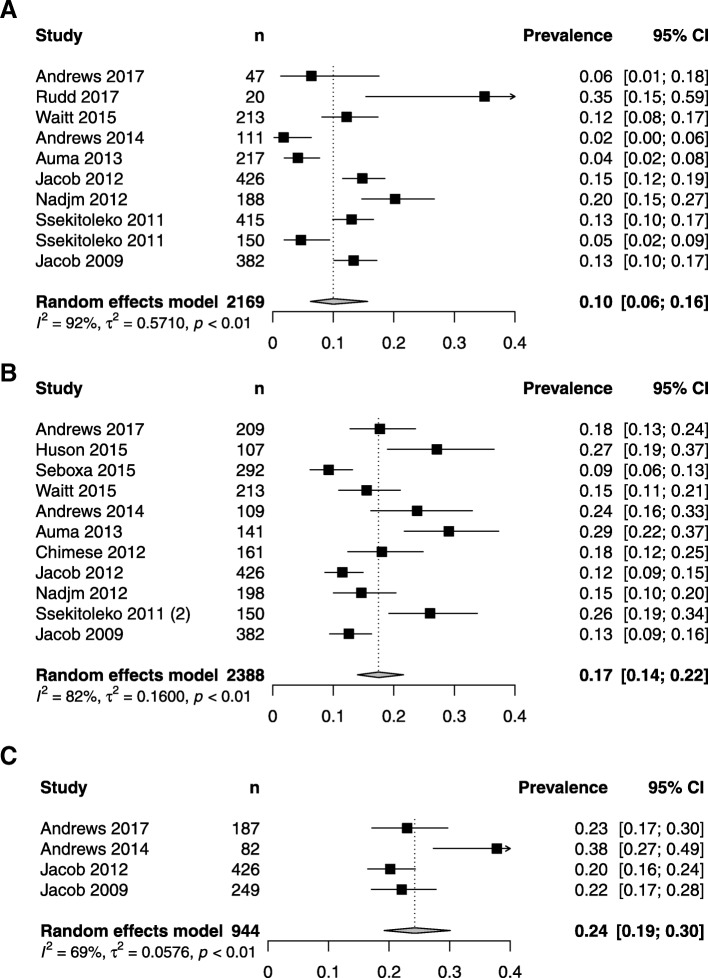
Pooled prevalence of **a** malaria, **b** bloodstream infection, and **c** *M. tuberculosis* bloodstream infection. Random effects summary estimate from a generalised linear mixed model is shown as well as measures of heterogeneity: *τ*^2^, *I*^2^, and *p* value from Cochran’s *Q* test

Aetiology data could be extracted from 14 studies: 11/14 reported aerobic blood culture data, 4/14 mycobacterial blood culture and 9/14 malaria data (Fig. 4), and 1/14 reported retrospective polymerase chain reaction (PCR) diagnostics on stored samples from Jacob et al. 2012 [34]. The data shown in Fig. 4 are those from the original Jacob et al. 2012 study [34] and not the retrospective PCR diagnostics; the latter are described below. Generally, studies performed aetiologic testing on all patients rather than restricting to a subgroup, excepting mycobacterial blood culture (carried out in four studies) which was restricted to HIV-infected participants in 2/4 studies and to a single study site in 1/4 studies (details of aetiologic testing availability by study are shown in Additional file 4).

Diagnosis of malaria was made by smear in all studies except the retrospective PCR study; one [29] additionally used rapid diagnostic tests and malaria-specific PCR. Pathogenic organisms isolated from aerobic blood culture are shown in Additional file 5. The commonest isolated organisms were *Staphylococcus aureus* (105/458 [23%]), non-typhoidal *Salmonella* (75/458 [16%]), and *Streptococcus pneumoniae* (68/458 [15%]). All studies reporting blood culture results described the microbiologic methods used but only 6/11 reported external quality control procedures during the study period.

Data on antimicrobial sensitivity patterns of the aerobic blood culture isolates were available in only 2/11 studies. One study [5] gave limited details only, stating that 95% of *Salmonella* isolates were resistant to chloramphenicol and trimethoprim-sulfamethoxazole and that none of the *Staphylococcus aureus* samples were resistant to oxacillin. The second study provided more comprehensive details, but with very small numbers which limit the conclusions that can be drawn; of 20 isolated Gram-negative bacilli (16 *E. coli*, the remainder *Klebsiella, Salmonella*, and *Citrobacter spp*.), 9/20 (45%) were resistant to third-generation cephalosporins, and this was strongly associated with mortality. All patients with third-generation cephalosporin-resistant BSI died, compared with 1/11 (9%) of those with sensitive infections (*p* < 0.0001).

One study [23] reported the results of a multiplex PCR on cryopreserved blood specimens of 336/426 participants and identified at least one potential pathogen in 245/336 samples, most commonly cytomegalovirus (139/336 [41%]), MTB (70/336 [21%]), *Plasmodium* spp. (35/336 [10%]), and *Streptococcus pneumoniae* (31/226 [9%]). Bacterial zoonoses or rickettsioses were uncommon (6/336 [2%]).

## Discussion

In-hospital sepsis and severe sepsis mortality in sSA is high. We found pooled in-hospital mortality of 19% (95% CI 12–29%) for Sepsis-2-defined sepsis and 39% (95% 30–47%) for Sepsis-2-defined severe sepsis. Pooled 28-/30-day Sepsis-2-defined severe sepsis mortality was 54% (95% CI 37–70%). Some between-study heterogeneity in sepsis mortality is likely consequent upon heterogeneous inclusion criteria, and some variability may be explained by the proportion of participants who are HIV-infected. Direct comparison of these mortality estimates to outcomes in high-income settings is difficult as estimates of sepsis mortality often derive from administrative databases and are sensitive to coding strategies. A recent meta-analysis [2] of population level estimates in post-2003 high-income settings estimated pooled sepsis and severe sepsis 30-day mortality to be of 17% (95% CI 11–26%) and 26% (95% CI 20–33%), respectively. The large US patient cohort used to validate the qSOFA score [8] (*n* = 74,453) found considerably lower mortalities for sepsis (as defined by Sepsis-2), at 4%.

It is likely that the sepsis outcomes we describe for sSA are worse than in high-income settings. The reasons for this are likely to be multifactorial, including lack of resources. The association of HIV status with mortality highlights the likely role of HIV as a driver of poor sepsis outcomes in sSA. It is also possible that delays in presentation to hospital or delays in processes of care may contribute to high mortality. The majority of studies we have identified provide no data to address these hypotheses, though there is some suggestion that presentation to care may be delayed. In the two Zambian RCTs [33, 35], 66–74% patients with severe sepsis were unable to walk on admission, and this had been the case for a median of 5–16 days; in Uganda, patients with severe sepsis had been unwell for a median on 14 days before arrival at hospital [24]. Data on processes of care are also largely lacking, though delays are apparent in one study: in the “before” arm of the before-after intervention trial in Uganda [5, 34], 49% (117/245) of patients with severe sepsis received no antimicrobials within 6 h of presentation, and a median of 500 ml of intravenous fluids was delivered in 6 h. However, in the usual-care arm of the two Zambian RCTs identified, mortality was high despite a prompt initiation of care: median door-to-antibiotic time was 1.3–1.5 h and a median 1.3–2.0 L of intravenous fluid was administered in the first 6 h of hospital admission. Lack of critical care facilities may play a role; again, data are largely absent, but across the two RCTs, only 1% (3/318) of patients were cared for on an ICU.

Of note, the 30-day mortality of severe sepsis seemed to be higher than the in-hospital mortality, though with overlapping confidence intervals. The reasons for this are not addressed in our study, but could represent uncontrolled primary infection, secondary infection following sepsis-related immunosuppression, non-infection-related mortality, or hospital practices such as palliative discharge of patients with poor prognosis. None of the studies we identified characterised longer-term (post 30-day) sepsis outcomes in sSA, and this highlights a major area for future research in resource-limited settings. Longer-term follow-up of patients in resource-limited settings is logistically difficult, as is matching episodes of care in administrative or clinical databases for individuals across fragmented and under-resourced health systems, which would be necessary to characterise sepsis outcomes from such databases; these factors may contribute to the lack of available data. No identified study characterised morbidity or economic cost either to health systems or individuals, but the combination of poor outcomes in a young adult population—often the most economically productive group in any society—may have significant socio-economic implications.

In contrast to high-income settings, the populations recruited to the identified studies are young and predominantly HIV-infected. It is likely that the high HIV prevalence influences the causative pathogens: the commonest cause of bloodstream infection, when sought, was *M. tuberculosis*, and non-typhoidal Salmonellae and *S. pneumoniae* were also commonly isolated, both of which have a strong association with HIV [36, 37]. Management of HIV in the critically ill in LMICs is poorly described; this review highlights that a carefully considered strategy for HIV must be developed and that data to inform it are required. The high prevalence of *S. aureus* is perhaps surprising, given data on incidence of causative agents of BSI in sSA [38]. Though this could represent true BSI, the possibility of a poor collection technique or laboratory misidentification should be considered; though it was possible to exclude coagulase-negative *Staphylococci* from our analysis as likely contaminants, determining whether *S. aureus* bacteraemia represented true BSI was not possible with the available data. Despite the fact that malaria diagnostic data is from studies carried out in malaria-endemic countries—Malawi, Uganda, Tanzania, and Zambia—malaria was less common than bacterial bloodstream infection, highlighting the emerging importance of non-malarial aetiology of fever in malaria-endemic areas. In the single study employing PCR to describe sepsis aetiology beyond bloodstream infection, CMV was extremely commonly isolated. While it may cause illness in the immunocompromised, CMV reactivation in critical illness is recognised in high-income settings. Without viral testing in a non-sepsis control group, the causal role of CMV is therefore not clear [39]. Bacterial zoonoses were rare, in contrast to sparse data from fever aetiology studies from elsewhere in sSA [18], though no study carried out reference-standard serological assays. Data on antimicrobial resistance (AMR) patterns were lacking: a single study with small numbers of patients, found that Gram-negative third-generation cephalosporins resistance (3GC-R) was significantly associated with mortality. This, alongside data from across the continent suggesting that 3GC-R is an emerging problem [38, 40, 41], highlights the need for high-quality national and sub-national surveillance for AMR across sSA to guide locally appropriate therapies.

The high prevalence of tuberculosis as a cause of sepsis has significant implications for appropriate antimicrobial therapy in sSA. Current sepsis protocols in high-income settings are based on rapid administration of broad-spectrum antimicrobials to cover common Gram-positive and Gram-negative pathogens, rather than Mycobacteria. However, it is far from clear how tuberculosis therapy should be used in this patient population in sSA, and due to lack of data, it is not possible to make any recommendations from the studies we identified. Further studies are needed to guide clinicians in the best use of tuberculosis diagnostic tests or empiric therapy in sepsis, as are pharmacokinetic studies of tuberculosis therapy in the critically unwell.

We identified relatively few studies. Existing studies were geographically restricted: Uganda contributed 8/15 studies, for example, and Zambia 3/15. Several institutions—largely tertiary referral centres—contributed multiple studies. Most studies excluded surgical or obstetric patients. The studies largely used a Sepsis-2 definition of sepsis and severe sepsis, though with a number of modifications, making between-study comparisons difficult. Development of easily applicable sepsis definitions for low-resource settings—or pragmatic modification of currently used screening scores such as qSOFA—could significantly increase the generalisability and utility of the evidence base in sSA.

There are weaknesses to our review, including that our searches may have missed studies. We excluded studies in preselected populations (in particular obstetric or surgical sepsis) to maximise the generalisability of our findings and minimise potential bias. These populations were also excluded a priori by identified studies, leading to lack of generalisability to surgical or pregnant patients. Much of sSA is unrepresented, potentially leading to bias from geographical restriction. Similarly, the results may not generalise to sepsis in district hospitals, or primary health care facilities, given the predominance of studies from tertiary facilities. The heterogeneity in mortality estimates means that the summary estimates should be regarded with caution. As with all meta-regression, there is the possibility of confounding or ecologic bias. Where it was not possible to disaggregate data from identified studies, we did not attempt to access individual participant-level data. Assessing risk of bias for observational studies is difficult, with no recognised gold standard. We chose to use the Newcastle-Ottawa score, a frequently used tool, but one which has recognised problems with reliability and validity [42, 43] and as such our estimates of bias are likely to be under-estimates.

## Conclusions

We have demonstrated that sepsis in sSA is dominated by the high prevalence of advanced HIV, subjecting a younger population to a high risk of sepsis of different microbial aetiology to high-income/low-HIV settings. Short-term outcomes are poor despite the younger age of sepsis patients compared to high-income settings, whilst long-term outcome data and morbidity data are absent. Data on sepsis aetiology beyond bloodstream infection and malaria are lacking though tuberculosis clearly plays an important role, which has implications for selection of appropriate antimicrobial therapy, though further studies are needed to develop an evidence base for the treatment of tuberculosis in the critically ill. Development and deployment of easily applicable sepsis definitions would help improve our understanding of the burden of sepsis in sSA and intervention studies aimed at the distinct population in sSA are urgently needed.

Box 1: Systematic review search terms Sepsis AND ((Angola OR Benin OR Botswana OR Burkina Faso OR Burundi OR Cameroon OR Cape Verde OR Central African Republic OR Chad OR Comoros OR Republic of the Congo OR Congo Brazzaville OR Democratic republic of the Congo OR Cote d’Ivoire OR Djibouti OR Equatorial Guinea OR Eritrea OR Ethiopia OR Gabon OR The Gambia OR Ghana OR Guinea OR Guinea-Bissau OR Kenya OR Lesotho OR Liberia OR Madagascar OR Malawi OR Mali OR Mauritania OR Mauritius OR Mozambique OR Namibia OR Niger OR Nigeria OR Reunion OR Rwanda OR Sao Tome and Principe OR Senegal OR Seychelles OR Sierra Leone OR Somalia OR South Africa OR Sudan OR Swaziland OR Eswatini OR Tanzania OR Togo OR Uganda OR Western Sahara OR Zambia OR Zimbabwe) OR Africa)

## Additional files

Additional file 1: Modified Newcastle-Ottawa Quality Assessment Scale. (PDF 31 kb)

Additional file 2: Results of quality assessment using modified Newcastle-Ottawa scale. Numbers are a normalised score for a given domain between 0 (low quality) and 1 (high quality). (PDF 5 kb)

Additional file 3: Inclusion and exclusion criteria for included studies. (DOCX 16 kb)

Additional file 4: Availability of diagnostic testing by study. (DOCX 15 kb)

Additional file 5: Species of pathogenic bacteria isolated from aerobic blood culture. NTS = non-typhoidal Salmonellae (PDF 5 kb)

## Data Availability

Not applicable.

## Abbreviations

AMR: Antimicrobial resistance
BSI: Bloodstream infection
CD4: Cluster of differentiation 4
HIV: Human immunodeficiency virus
PCR: Polymerase chain reaction
PRISMA: Preferred Reporting Items for Systematic Reviews and Meta-Analyses
qSOFA: Quick sequential organ failure assessment
SIRS: Systemic inflammatory response score
SOFA: Sequential organ failure assessment
sSA: Sub-Saharan Africa
