# Supplementary material for: Aetiology and outcomes of sepsis in adults in sub-Saharan Africa: a systematic review and meta-analysis
Source: Crit Care. 2019 Jun 11;23:212. doi: 10.1186/s13054-019-2501-y (PMC6558702; doi:10.1186/s13054-019-2501-y)
Supplement: Supplementary file 1 — Modified Newcastle-Ottawa Quality Assessment Scale. (PDF 31 kb) [file 13054_2019_2501_MOESM1_ESM.pdf]

MODIFIED NEWCASTLE - OTTAWA QUALITY ASSESSMENT SCALE
COHORT STUDIES

Note: A study can be awarded a maximum of one star for each numbered item within the Selection and Outcome categories. A maximum of two stars can be given for Comparability

Selection

1) Representativeness of the exposed cohort
a) truly representative of the average adult with sepsis in the community ❋
b) somewhat representative of the average adult with sepsis in the community ❋
c) selected group of users eg nurses, volunteers
d) no description of the derivation of the cohort

2) Ascertainment of exposure – HIV status
a) reproducible description of valid testing procedures (e.g. following national guidelines)❋
b) self reported status
c) no description

3) Ascertainment of exposure - aetiology
a) Reproducible description of testing procedures using reference tests ❋
b) Reproducible description of testing using non-reference tests (e.g Widal test for enteric fever)
d) no or nonreproducible description of testing

Comparability

1) Comparability of cohorts on the basis of the design or analysis
a) study reports +/- controls for lactate ❋
b) study reports +/- controls for any additional physiologic parameters (BP, HR etc) ❋

Outcome

1) Assessment of outcome

a) independent blind assessment ✵

b) record linkage ✵

c) self report

d) no description

2) <u>Adequacy of follow up of cohorts</u>

a) complete follow up - all subjects accounted for ✵

b) subjects lost to follow up unlikely to introduce bias - small number lost - > 90% follow up rate, or description provided of those lost) ✵

c) follow up rate < 90% and no description of those lost

d) no statement
