# Supplementary material for: Aetiology and outcomes of sepsis in adults in sub-Saharan Africa: a systematic review and meta-analysis
Source: Crit Care. 2019 Jun 11;23:212. doi: 10.1186/s13054-019-2501-y (PMC6558702; doi:10.1186/s13054-019-2501-y)
Supplement: Supplementary file 2 — Results of quality assessment using modified Newcastle-Ottawa scale. Numbers are a normalised score for a given domain between 0 (low quality) and 1 (high quality). (PDF 5 kb) [file 13054_2019_2501_MOESM2_ESM.pdf]

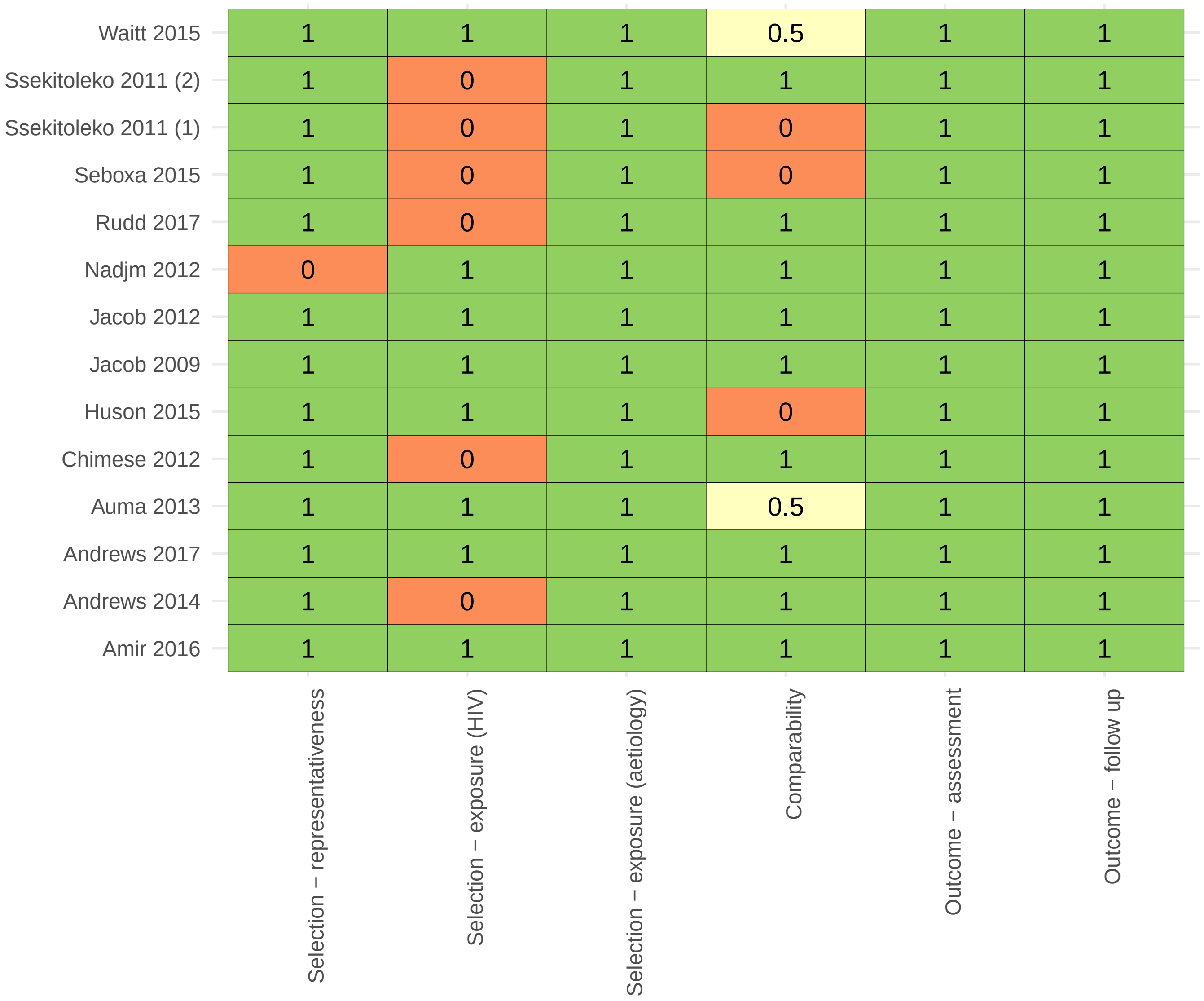
Waitt 2015 1 1 1 0.5 1 1
Ssekitoleko 2011 (2) 1 0 1 1 1 1
Ssekitoleko 2011 (1) 1 0 1 0 1 1
Seboxa 2015 1 0 1 0 1 1
Rudd 2017 1 0 1 1 1 1
Nadjm 2012 0 1 1 1 1 1
Jacob 2012 1 1 1 1 1 1
Jacob 2009 1 1 1 1 1 1
Huson 2015 1 1 1 0 1 1
Chimese 2012 1 0 1 1 1 1
Auma 2013 1 1 1 0.5 1 1
Andrews 2017 1 1 1 1 1 1
Andrews 2014 1 0 1 1 1 1
Amir 2016 1 1 1 1 1 1
Selection – representativeness
Selection – exposure (HIV)
Selection – exposure (aetiology)
Comparability
Outcome – assessment
Outcome – follow up
