# Supplementary material for: Aetiology and outcomes of sepsis in adults in sub-Saharan Africa: a systematic review and meta-analysis
Source: Crit Care. 2019 Jun 11;23:212. doi: 10.1186/s13054-019-2501-y (PMC6558702; doi:10.1186/s13054-019-2501-y)
Supplement: Supplementary file 3 — Inclusion and exclusion criteria for included studies. (DOCX 16 kb) [file 13054_2019_2501_MOESM3_ESM.docx]

| Study | Inclusion criteria | Exclusion criteria |
| --- | --- | --- |
| Jacob 2009 | (1) 18+ years in daytime hours, susp. infection (2) 2 or more of axillary T > 37.5C or < 35.5C, HR > 90, RR > 20 (3) SBP < 100 | acute CVA, GI haemorrhafe, required admission to nonmedical ward, unable to consent and no guardian |
| Nadjm 2012 | (1) “adult” wed-sun, (2) fever or history or fever, (3) any one of confusion/coma, deep breathing, severe tachypnoea or hypoxia, severe pallor, visible jaundice, unable to walk, diagnosis of "severe malaria" | none |
| Jacob 2012 and Moore 2018 | (1) 18+ years in daytime hours, susp. infection (2) 2 or more of axilliary T > 37.5C or < 35.5C, HR > 90bpm, RR > 20bpm (3)  SBP < 100mmHg, (4)  lactate > 2.5mmol/L or KPS <= 40, (5) available guardian | suspected surgical or obstetric emergencies or shock without suspected infection |
| Waitt 2015 | ( 1)16+ years Mon-Fri, susp infection, (2) 2 or more of T > 38.3C or < 35C, RR > 20bpm, HR > 90bpm, WCC > 12,000/µL or < 4,000/µL, altered mental state | hospitalised or recieved antibiotics in last 2 weeks, unable to consent (ie obtunded and no guardian) |
| Ssekitoleko 2011 (1) | (1) 18+ yrs , suspected infection, (2) 2 or more of axillary T of > 37.5°C or <35.5°C, HR > 90, RR> 20, (3) SBP < 100mmHg, (4) one of lactate of >4 mmol/L, platelet number of <100,000/µL, or altered mental state | acute cerebrovascular event or gastrointestinal hemorrhage or needed triage to a surgery or obstetrics and gynecology ward |
| Ssekitoleko 2011 (2) | 18+ yrs Mon-Fri; (2) 18year+; (3) at least 2 of T < 36C or > 38C, RR > 20bpm, HR > 90bpm | Required triage to surgical or obs&gynae ward, had recieved any treatment or IV fluids before recruitment or suspected CVA or gastrointestinal haemorrhage |
| Chimese 2012 | 1) 16+ years, susp. infection 2) 2 of T > 38C or < 36C, HR > 90bpm, RR > 20bpm, WCC > 12,000/µL or < 4,000/µL | none |
| Andrews 2014 | (1) 18+ year Mon-Fri, susp. infection, 2) 2+ of HR> 90bpm, RR > 20bpm, T >=38.0C or <=36.0C, or WBC > 12,000/µL or < 4,000/µL, and 3) One of SBP < 90 mmHg or MAP<65mmHg, altered mentation, creatinine > 1.85 mg/dL, platelet count<100,000/µL, resp>40, jaundice | gastrointestinal bleed, requiredimmediate surgery, congestive heart failure exacerbation or end-stage renal disease or raised jugular venous pressure (JVP) > 3 cm above the sternal angle, as measured with a level and a ruler. |
| Auma 2013 | (1) 18+ medical admission 08:00-00:00 susp. infection (2) 2+ of axillary T>37.5 or T<35.5, HR>90bpm, RR>20bpm | surgical, o&g admission |
| Andrews  2017 | (1) 18+ year Mon-Fri, susp. infection, 2) 2+ of HR> 90bpm, RR > 20bpm, T >=38.0C or <=36.0C, or WBC > 12,000/µL or < 4,000/µL, and 3) SBP < 90 mmHg or MAP<65mmHg | RR > 40 or SpO2 < 90%, gastrointestinal bleed, requiredimmediate surgery, congestive heart failure exacerbation or end-stage renal disease or raised jugular venous pressure (JVP) > 3 cm above the sternal angle, as measured with a level and a ruler. |
| Huson 2014 | 1) 18+ years, clinical evidence of infection, weekdays 2) tympanic T >= 38C or < 36C, 3) one of HR > 90bpm, RR > 20bpm, WBC > 12,000/µL or < 4,000/µL | none |
| Seboxa 2015 | 1) "Adult" 2) 2+ of axilliary T > 38.5 or < 36.5, HR > 90bpm, RR > 20bpm | Received antimicrobials within last 72hr |
| Rudd 2017 | 1) 18+ years 2) 2+ of T > 38 or < 36, HR > 90bpm, RR > 20bpm or PCO2 < 32mmHg, WBC > 12,000/µL or < 4,000/µL or > 10% band forms | Surgical patients, admission to maternity ward |
| Amir 2016 | (1) 14+ years, susp. infection; (2) 2+ of axillary T >= 38°C or< 36 36°C, HR > 90 bpm, RR > 20bpm, or WBC > 12,000/µL or < 4,000/µL, (3) signs of end-organ dysfunction including SBP <= 90 platelet count<100,000/µL or GCS<15 | Triage to a surgical or o&g ward, had received any antibiotics or intravenous fluids prior to recruitment, or had a history suggestive of other diagnoses associated with lactic acidosis such as diabetic ketoacidosis, acute coronary syndrome, or chronic liver disease |

Supplementary table 1: inclusion criteria for studies.
