# Supplementary material for: Aetiology and outcomes of sepsis in adults in sub-Saharan Africa: a systematic review and meta-analysis
Source: Crit Care. 2019 Jun 11;23:212. doi: 10.1186/s13054-019-2501-y (PMC6558702; doi:10.1186/s13054-019-2501-y)
Supplement: Supplementary file 4 — Availability of diagnostic testing by study. (DOCX 15 kb) [file 13054_2019_2501_MOESM4_ESM.docx]

| **Study** | **Total number of participants** | **Available aetiology data** | | |
| --- | --- | --- | --- | --- |
|  |  | **Blood culture** | **Mycobacterial blood culture** | **Malaria** |
| Jacob 2009 | 382 | 382/382 (100%) | 249/382* (65%) | 382/382 (100%) |
| Nadjm 2012 | 198 | 198/198 (100%) | ND | 188/198 (95%) |
| Jacob 2012 | 426 | 426/426 (100%) | 426/426 (100%) | 426/426 (100%) |
| Waitt 2015 | 213 | 213/213 (100%) | ND | 213/213 (100%) |
| Ssekitoleko 2011 (1) | 96 | NR | NR | NR |
| Ssekitoleko 2011 (2) | 150 | 150/150 (100%) | ND | 150/150 (100%) |
| Chimese 2012 | 161 | 161/161 (100%) | ND | ND |
| Andrews 2014 | 112 | 103/112 (92%) | 82/112* (73%) | 109/112 (92%) |
| Auma 2013 | 216 | 141/216 (65%) | ND | 216/216 (100%) |
| Andrews 2017 | 209 | 209/209 (100%) | 187/209* (89%) | 47/209 (22%) |
| Huson 2014 | 107 | 107/107 (100%) | NR | NR |
| Seboxa 2015 | 292 | 292/292 (100%) | ND | ND |
| Rudd 2017 | 20 | ND­ | ND | 20/20 (100%) |
| Amir 2016 | 218 | ND | ND | ND |
|  |  |  |  |  |

Supplementary Table 2: available aetiology data by study. * = mycobacterial blood cultures were carried out at one study site only; ** = mycobacterial blood cultures were carried out in HIV positive participants only. One study (Moore 2018) which carried out retrospective testing of stored samples from Jacob 2012 is not included in this table, but is described in the text.
