# Supplementary figures and images for: Aetiology and outcomes of sepsis in adults in sub-Saharan Africa: a systematic review and meta-analysis
Source: Crit Care. 2019 Jun 11;23:212. doi: 10.1186/s13054-019-2501-y (PMC6558702; doi:10.1186/s13054-019-2501-y)

Organism

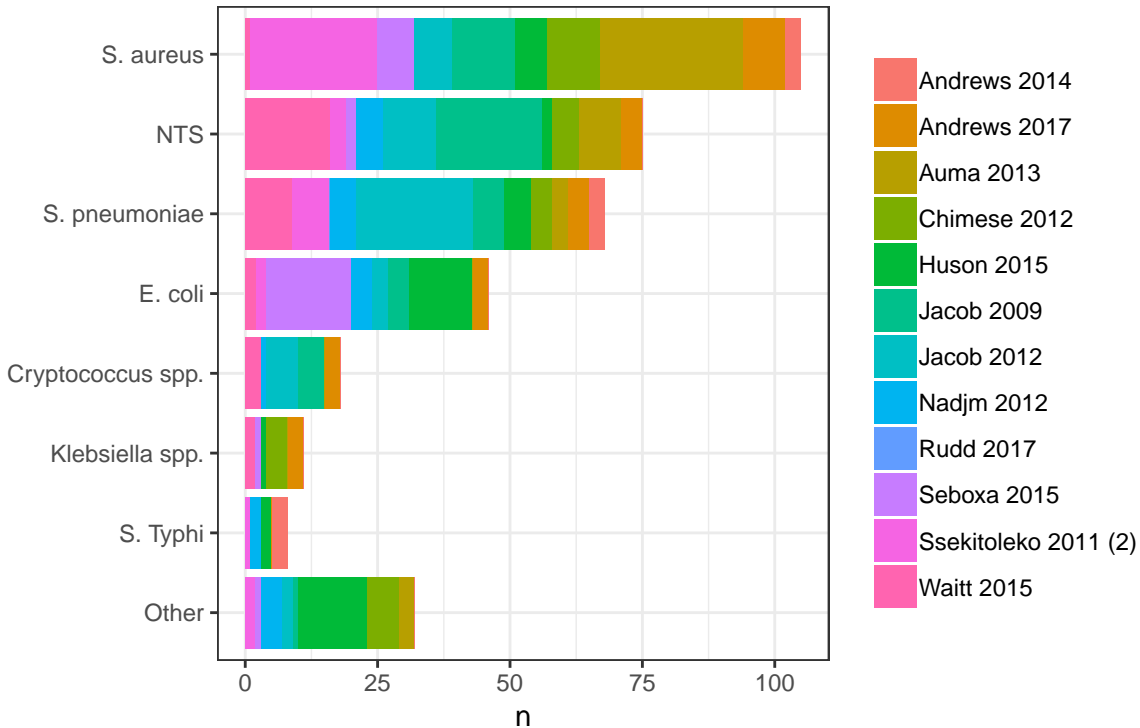

Supplement: Supplementary file 5 — Species of pathogenic bacteria isolated from aerobic blood culture. NTS = non-typhoidal Salmonellae (PDF 5 kb) [file 13054_2019_2501_MOESM5_ESM.pdf]
